# Network pharmacology combined with molecular docking and experimental validation of the mechanism of action of columbianetin acetate in the treatment of ovarian cancer

**DOI:** 10.3389/fonc.2025.1515976

**Published:** 2025-02-25

**Authors:** Mengling Hu, Luyao Wang, Feiyue Zhang, Yiluo Xie, Tingting Zhang, Hongli Liu, Zhenghong Li, Jing Zhang

**Affiliations:** ^1^ Department of Genetics, School of Life Sciences, Bengbu Medical University, Bengbu, China; ^2^ Department of Clinical Medicine, Bengbu Medical University, Bengbu, China; ^3^ Department of Gynecological Oncology, First Affiliated Hospital of Bengbu Medical University, Bengbu, China

**Keywords:** network pharmacology, columbianetin acetate, ovarian cancer, metastasis, apoptosis

## Abstract

**Background:**

Ovarian cancer is the most prevalent malignant tumor of the female reproductive system and has the highest mortality rate among gynecological cancers. Columbianetin acetate (CE) is one of the active ingredients of Angelica sinensis, which has good antifungal and anti-inflammatory activities. However, its potential mechanism of action in ovarian cancer remains unclear. This study used network pharmacology and molecular docking technology to investigate the molecular mechanism and material basis of CE in the treatment of ovarian cancer, and further verified by *in vitro* experiments.

**Methods:**

Relevant targets for CE were obtained from TCMSP and SwissTargetPrediction databases. OMIM, GeneCards and DisGeNET databases were applied to screen ovarian cancer-related targets. The STRING database to obtain protein-protein interaction (PPI) network. Then key targets were obtained using Cytoscape software, followed by expression, survival and ROC diagnostic analyses of core genes using R software. GO and KEGG enrichment analyses were performed using the DAVID database. Binding ability of CE to core targets was assessed by molecular docking. KEGG sites were used to predict core gene-related pathways. Subsequently, *in vitro* cellular experiments were performed to further investigate the molecular mechanism of CE treatment for ovarian cancer.

**Results:**

A total of 55 CE-ovarian cancer interaction targets were identified using network pharmacology techniques. Among these, eight key targets —ESR1, GSK3B, JAK2, MAPK1, MDM2, PARP1, PIK3CA, and SRC—were screened using Cytoscape software. Core genes ESR1, GSK3B and JAK2 were obtained based on expression, prognostic and diagnostic values using R software. GO and KEGG enrichment analyses indicated that CE treatment of ovarian cancer might be related to PI3K/Akt signaling pathway, MAPK signaling pathway, ErbB signaling pathway and Ras signaling pathway. The molecular docking results showed that CE had good binding ability with core targets ESR1, GSK3B and JAK2. The results of *in vitro* cellular experiments indicated that CE may inhibit the proliferation and metastasis of ovarian cancer and promote apoptosis by inhibiting the PI3K/AKT/GSK3B pathway.

**Conclusions:**

Based on the network pharmacology approach, we predicted the potential mechanism of CE for the treatment of ovarian cancer, which provided a new idea for further research on its pharmacological mechanism.

## 1 Introduction

One of the main causes of death for women worldwide is ovarian cancer. An estimated 313,000 new cases and 152,000 deaths globally are attributed to ovarian cancer each year (1). In the United States, ovarian cancer ranks fifth among cancer-related fatalities among women, and globally, it is the eighth most common cause of death for women (2). It is commonly recognized that the main course of treatment for ovarian cancer involves surgery as the primary intervention, adjuvant chemotherapy as a supplement, and a focus on a mix of therapeutic techniques (3, 4). However, because cancer cells have a poor susceptibility to drugs and a high level of resistance, existing treatments typically fail (5). Therefore, the search for new treatment modalities for ovarian cancer is urgent.

Traditional Chinese medicine (TCM) and their derivatives are the most represented alternative treatment for resolving health issues, including cancer, according to accumulating research (6–9). The benefits of traditional Chinese medicine (TCM) include its ability to address various targets, few side effects, and high curative efficacy (10). Columbianetin acetate (CE) is one of the main active constituents isolated from Angelica sinensis, which has been demonstrated to have a range of biological effects, such as antioxidation and anti-inflammatory properties (11). Unfortunately, there are very few reports on CE and even fewer studies on the mechanisms of CE in cancer. Therefore, the present study mainly used network pharmacology and molecular docking techniques to try to explore the molecular mechanism of the effect of CE on ovarian cancer.

**Figure 1:**
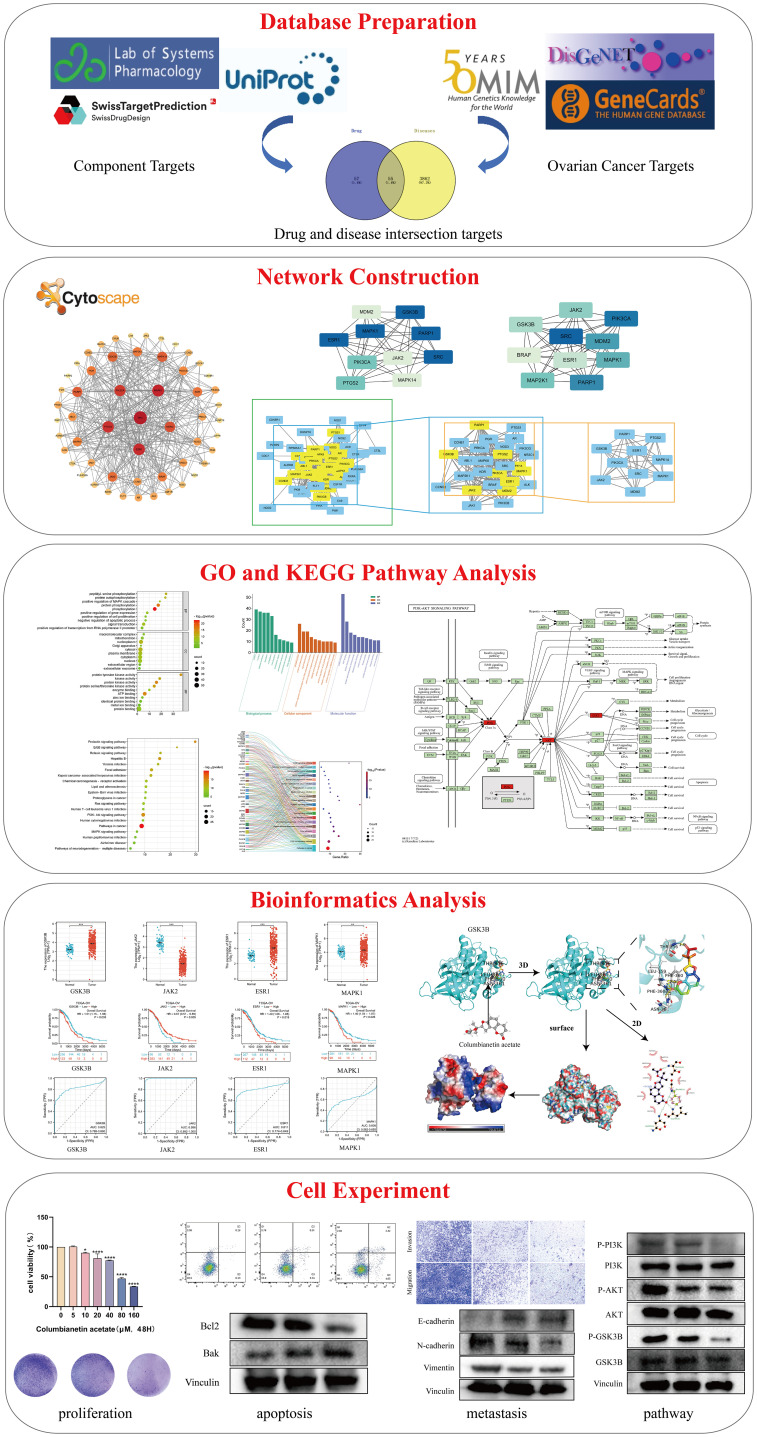
Flowchart of this study.

The paradigm for medical research has gradually shifted as interdisciplinary subjects like bioinformatics, systems biology, and computational biology have grown in popularity. The research strategy of disease diagnosis and treatment has also changed from “single disease, single target, single drug” to “multi-target, systematic regulation” (12, 13). Network pharmacology is a new interdisciplinary discipline based on systems biology, genomics, proteomics, etc., which uses computer to integrate a large amount of information and discover new drug targets and molecular mechanisms (14). This is a useful instrument for obtaining a full and methodical understanding of the workings of multi-ingredient medication from a holistic perspective (15). Therefore, in the present study, we investigated the molecular mechanism of CE in the treatment of ovarian cancer, starting from the prediction of network pharmacology, and performed *in vitro* experiments for further validation. These findings provide new insights into the active ingredients of traditional Chinese medicine for the treatment of cancer and provide theoretical guidance for further clinical applications. The flowchart of this study is shown in **Figure 1**.

## 2 Materials and methods

### 2.1 Collecting potential targets for ovarian cancer

Using “ovarian cancer” as the keyword, potential therapeutic targets for ovarian cancer were retrieved from the Online Mendelian Inheritance in Man (OMIM) database (https://omim.org/), the DisGeNET database (https://www.disgenet.org/), and GeneCards database (http://www.genecards.org/).

### 2.2 Collection of CE-related targets

By using Traditional Chinese Medicine Systematic Pharmacology Database and Analysis Platform (TCMSP, http://lsp.nwu.edu.cn/tcmsp.php), Swiss Target Prediction database (http://www.swisstargetprediction.ch) to obtain the targets corresponding to CE, and the target information was standardized through the Universal Protein Resource Database (UniProt, https://www.uniprot.org/). The standardized targets were merged and duplicates were removed to obtain CE-related targets.

### 2.3 Construction of a common target PPI network for CE and ovarian cancer

Importing targets that intersect with drugs and diseases into the STRING database (http://string-db.org). In the operator interface, the species was limited to “Homo sapiens” and the confidence level was set to ≥ 0.7 to generate the PPI network. The results were visualized by Cytoscape 3.10.1 software, and the network topology parameters of these targets were obtained using the tool Analysis network in Cytoscape, with the size of the nodes and the shade of the color adjusted according to the degree value, and the plug-ins cytoHubba and CytoNCA in Cytoscape software were also used to further screen the core targets.

### 2.4 GO and KEGG enrichment analyses

CE and ovarian cancer crossover genes were imported into the DAVID (https://david.ncifcrf.gov/) database for gene ontology (GO) annotation and Kyoto Encyclopedia of the Genome (KEGG) enrichment analysis. GO analysis was performed in terms of biological process (BP), molecular function (MF), and cellular component (CC). The obtained data were uploaded to the bioinformatics (http://www.bioinformatics.com.cn/) platform for visualization and analysis.

### 2.5 Screening of core genes based on clinical value

To understand the clinical significance of the key genes, we integration of gene expression data of ovarian cancer and normal ovarian epithelial tissues from TCGA database (https://portal.gdc.cancer.gov/) and GTEx database (https://commonfund.nih.gov/GTEx), using unified RNA-seq data in TPM format from TCGA and GTEx databases. Based on the clinical information of these samples, differential analysis, Kaplan-Meier analysis and ROC diagnosis were performed using the stats package, car package, survival package and pROC package in R 4.4.1 software, and the results were all visualized using ggplot2 package.

### 2.6 Molecular docking validation

Based on the above analysis, we validated the molecular docking of CE with core targets. The 3D crystal structures of the target proteins were obtained in the Protein Data Bank (PDB, https://www.rcsb.org/). The corresponding PDB format files were downloaded, water molecules, ions, and ligands were removed using PyMOL software, and the processed proteins were saved as PDBQT files. The mol2 files of CE were obtained through TCMSP database (http://lsp.nwu.edu.cn/tcmsp.php), hydrogenated using AutoDockTools 1.5.7 software, charge numbers were calculated, molecular rigidity properties were determined and exported as PTBQT files. After that, molecular docking was performed by AutoDock Vina to predict the binding energy between CE and the core target, and 3D visualization was performed using PyMOL software, and 2D visualization was performed by LigPlot software.

### 2.7 Diagram of the pathway of CE action in ovarian cancer

Key targets were red-flagged by visualizing the PI3K/AKT signaling pathway, a key pathway for CE action in ovarian cancer, through the KEGG Mapper tool on the KEGG website (https://www.kegg.jp/).

### 2.8 Cell culture

The human ovarian epithelial cell line IOSE-80 was purchased from iCell Biosciences (Shanghai, China). Ovarian cancer cell lines SKOV3 and A2780 were purchased from the Shanghai Cell Bank of the Chinese Academy of Sciences and Starfish Biotechnology (Jiangsu, China). The medium were McCoy’s 5A medium and RPMI 1640 medium (Gibco, ThermoFisher Scientific, USA) supplemented with 10% fetal bovine serum (FBS) and 1% penicillin-streptomycin. Cells were placed in a humidified incubator at 37°C and 5% CO_2_. CE was purchased from MCE (Shanghai, China), dissolved in DMSO, and diluted to the appropriate concentration.

### 2.9 Cell proliferation capacity assay

Cell proliferation assay was performed using Cell Counting Kit-8 (CCK-8). IOSE-80, SKOV3 and A2780 cells were inoculated in 96-well plates at a density of 4 × 10^3^ cells/well. After overnight incubation in the incubator, the cells were treated with 0, 5, 10, 20, 40, 80 and 160 μM of CE for 48 h. After 48 h, 10 μL of CCK-8 solution (Bimake, USA) was added to each well, and the cells were incubated at 37°C for 2 h. The OD values were determined at 450 nm using an enzyme labeller (BioTek Inc., USA, cytation3, Inc.), and the IC50 concentration of CE was calculated for subsequent experiments. For colony formation experiment, 1× 10^3^ cells were inoculated in 6-well plates and cultured. After 48 h of CE treatment, the cells were fixed with 4% paraformaldehyde for 10 min, washed twice, and then stained with 1% crystal violet staining solution. Finally, the number of colonies was counted by ImageJ software.

### 2.10 Apoptosis detection

FITC Annexin V Apoptosis Detection Kit (BD, USA) was used for apoptosis detection. SKOV3 and A2780 cells were treated with different concentrations of CE for 48 h, washed twice with pre-cooled PBS, digested, collected, centrifuged, and after centrifugation resuspended with 500 μL 1× binding solution, 5 μL of FITC Annexin V and 5 μL of PI were added. Mixed with the resuspended cells, and incubated in the dark for 30 min at room temperature. Apoptosis was measured by flow cytometry (BD Biosciences, USA) within 1 h.

### 2.11 Invasion and migration detection

Transwell and wound healing assays were used to determine the effect of CE on the ability of ovarian cancer cells to invade and migrate. Transwell assays were performed on 24-well plates using Transwell^®^ permeable scaffolds (Corning Incorporated, USA). After 48 h of CE treatment on SKOV3 and A2780 cells, the cells were resuspended in serum-free medium at a density of 3 × 10^4^ cells/well. Invasion assays required pre-coating of the upper chamber with matrix gel, while migration assays were performed without matrix gel. Then 600 μL of medium containing serum was added to the lower chamber. After 48 h of incubation, the cells were stained with crystal violet. Detection of cell migration using the scratch method. SKOV3 and A2780 cells were inoculated into 6-well plates, and when the cell number reached 90% or more, a straight line was scraped with the tip of a 100 μL pipette and the cells were treated with CE. Images of the cells were taken with a microscope at the indicated times (0, 24 h). And the area of wound closure was measured using ImageJ software.

### 2.12 Western blot assay

After CE treatment for 48 h, proteins were extracted from the cells and their concentrations were measured using the BCA protein assay kit (Beyotime, Shanghai, China). Subsequently, the proteins(20~30 μg)were separated by sodium dodecyl sulfate-polyacrylamide gel electrophoresis (SDS-PAGE). The separated proteins were transferred to a PVDF membrane (Immobilon-P, Carlsbad, Ireland) and closed with 5% skimmed milk for 2 h. Then the membrane was incubated in a refrigerator at 4°C overnight. Primary antibodies used: E-Cadherin (ET1607-75, HUABIO), N-Cadherin (ET1607-37, HUABIO), Vimentin (GB111308, Servicebio), Bcl-2 (GB113375, Servicebio), Bak (3814S, Cell Signaling Technology), PI3K (60225, Proteintech), Phospho- PI3K (AF3242, Affinity), AKT (A5031, Bimake), Phospho-AKT (4058S, Cell Signaling Technology), Phospho-GSK3B (ET1607-60, HUABIO), GSK3B (ET1607-71, HUABIO), Vinculin (66305, Proteintech). The next day, the membrane was placed in diluted secondary antibody. Bands were visualized using ECL chemiluminescence detection.

### 2.13 Statistical analysis

Experimental data were expressed as mean ± SD. Statistical data were analyzed using GraphPad Prism 8.0 software. Differences between two groups were analyzed by t-test, and comparisons between multiple groups were analyzed by one-way analysis of variance (ANOVA). p < 0.05 was considered statistically significant.

## 3 Results

### 3.1 Acquisition of common targets for CE and ovarian cancer

**Figure 2:**
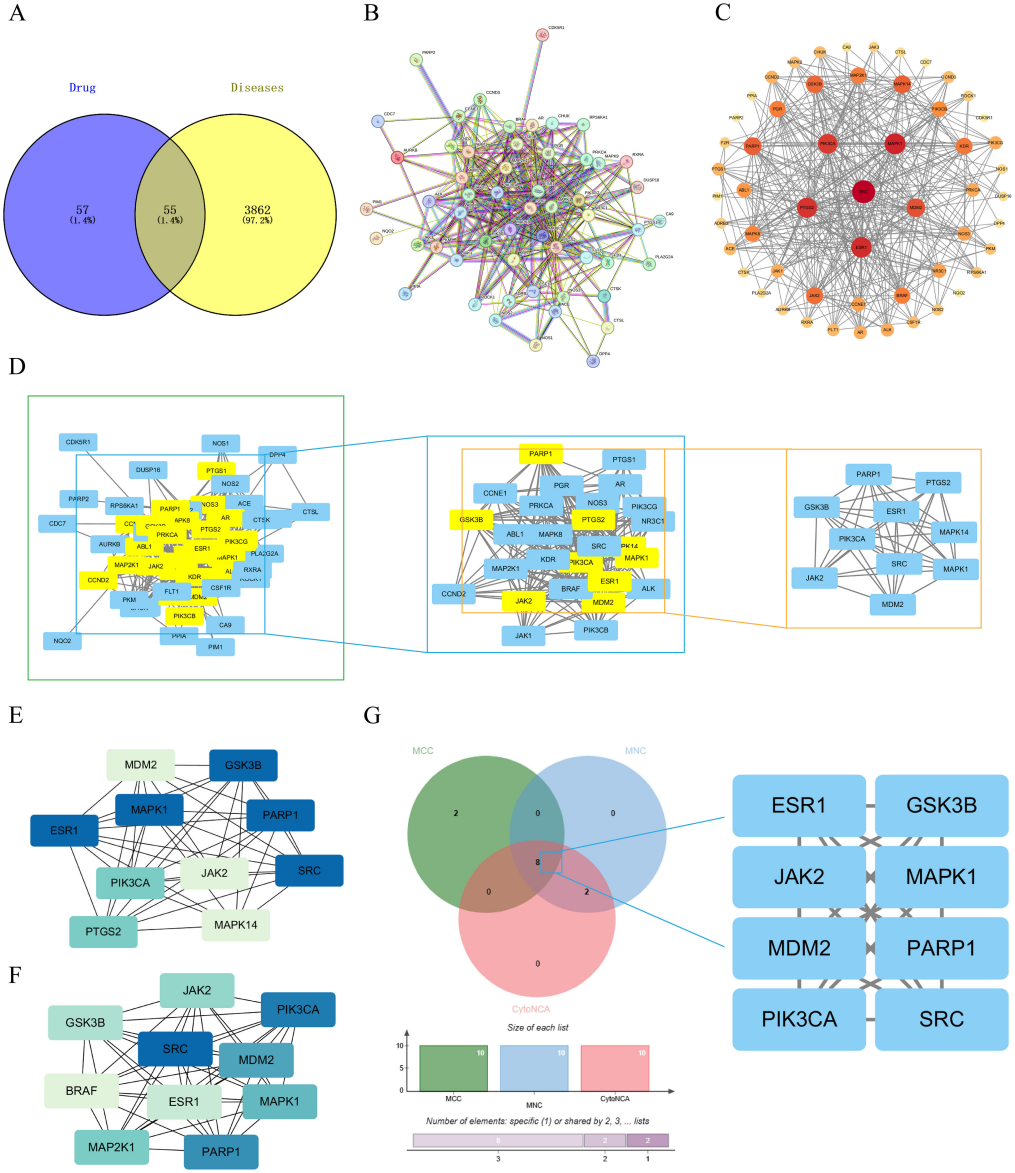
Acquisition of common targets between CE and ovarian cancer and construction of PPI networks. **(A)** Venn diagram of cross-targeting of CE and ovarian cancer. **(B)** PPI network diagrams available through the STRING database. **(C)** The PPI network graph is visualized through Cytoscape, where the size of the nodes is determined by the degree value. **(D)** Key targets were screened by CytoNCA selecting DC>22 (DC >2x median) as a criterion. **(E, F)** Key targets were screened using the MCC and MNC algorithms in the cytoHubba plugin. **(G)** The key targets obtained by the three algorithms are taken as intersections to obtain their crossover targets.

The CE targets were obtained from two databases, TCMSP and Swiss Target Prediction, with 14 targets obtained from TCMSP database and 100 targets from the Swiss Target Prediction database. The data from the two databases were combined and de-duplicated to obtain a total of 112 CE targets. Ovarian cancer targets were obtained from the OMIM, GeneCards and DisGeNET databases, 200 targets were obtained from the OMIM database, 2603 ovarian cancer targets were screened based on relevance scores ≥ 8 in GeneCards database, and 2563 relevant targets were screened based on scores ≥ 0.01 in DisGeNET database. The data from the three databases were combined and deduplicate, resulting in 3917 ovarian cancer-related targets. 55 overlapping targets between CE and ovarian cancer were obtained by Venny 2.1.0 (**Figure 2A**), and these 55 overlapping targets were used as possible therapeutic targets.

### 3.2 Construction of PPI network for CE and ovarian cancer

The 55 crossover targets were entered into the STRING website and the minimum interaction score was set to greater than 0.7 (**Figure 2B**). The data were then imported into Cytoscape software to construct a PPI network graph of CE interactions with ovarian cancer. There are 55 nodes and 365 edges in the network, The size and color of the nodes in the graph vary with the Degree value. The top six by Degree value are SRC, MAPK1, ESR1, PTGS2, PIK3CA, and MDM2 (**Figure 2C**). Next, using the CytoNCA plugin in Cytoscape software, a median DC value of 11 was used as the filtering condition, resulting in 29 nodes and 232 edges. When the DC was greater than twice the median (DC > 22), 10 nodes and 42 edges were obtained (**Figure 2D**). Meanwhile, the core genes were further screened using the Maximum Clonal Centrality (MCC) algorithm and Maximum Neighborhood Component (MNC) algorithm in Cytoscape’s plugin cytoHubba (**Figures 2E, F**). The key targets of CE acting on cells were further analyzed by combining the three Cytoscape software screening methods, and finally the 8 key targets of MDM2, PARP1, SRC, MAPK1, PIK3CA, ESR1, JAK2 and GSK3B were screened out (**Figure 2G**).

### 3.3 GO and KEGG pathway enrichment analysis

**Figure 3:**
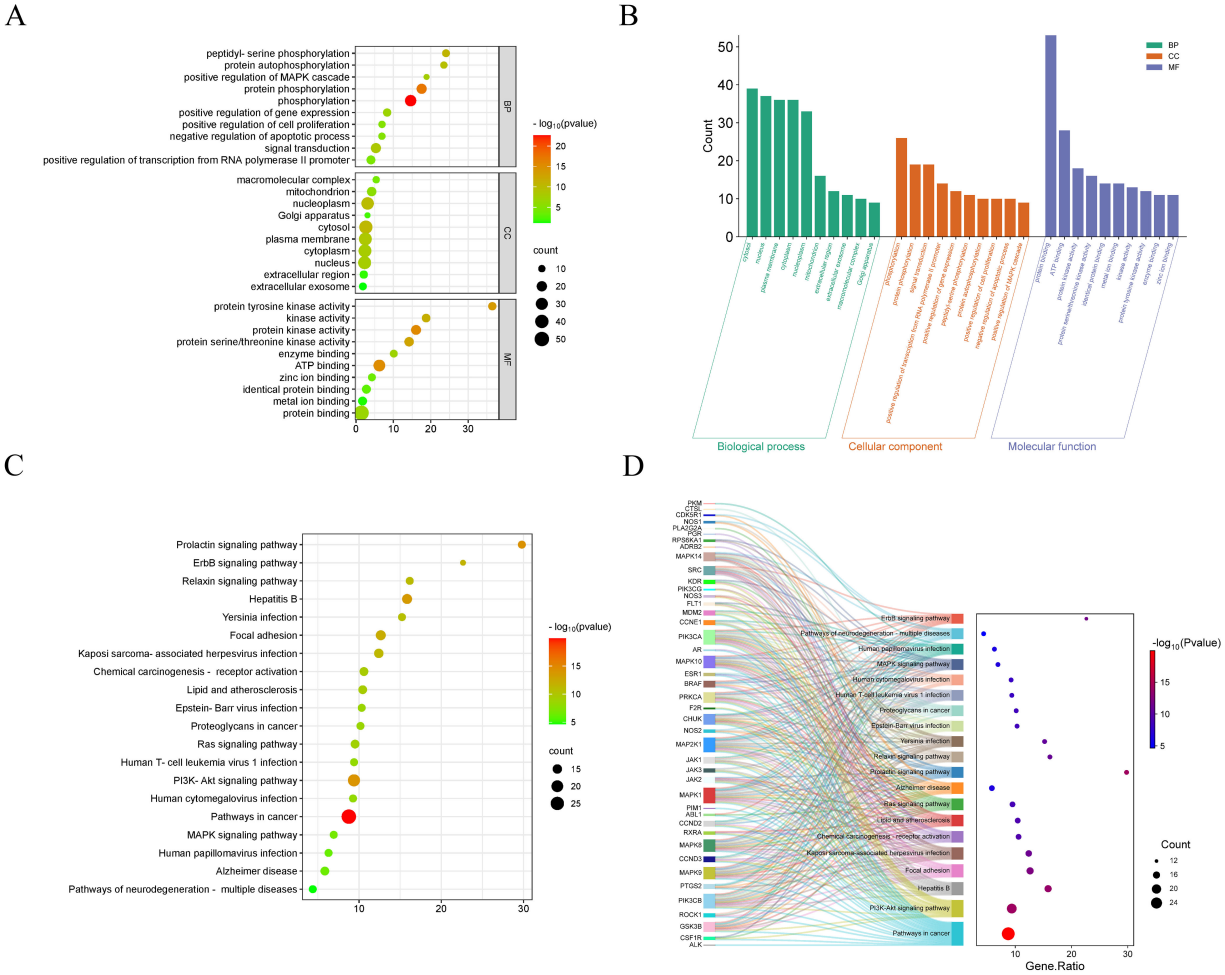
GO and KEGG enrichment analysis of CE-treated ovarian cancer. **(A, B)** Bubble and bar graphs for the first 20 items of Biological Processes (BP), Cellular Components (CC), and Molecular Functions (MF). **(C, D)** KEGG enrichment was demonstrated with bubble plots and Sankey plots of genes in relation to the top 20 pathways.

To further explore the molecular mechanism of CE treatment for ovarian cancer, we analyzed 55 intersecting genes for GO and KEGG enrichment. In the GO enrichment analysis, three items of biological process (BP), molecular function (MF) and cellular component (CC) were obtained. The top 10 items enriched for BP, CC and MF are represented by bubble and bar graphs (**Figures 3A, B**). BP is mainly associated with peptidyl-serine phosphorylation, positive regulation of MAPK cascade, protein phosphorylation, and signal transduction. CC is mainly related to macromolecular complexes, mitochondria, extracellular regions, cellular exosomes, etc. MF is mainly related to protein tyrosine kinase activity, protein serine/threonine kinase activity, and so on. Subsequently, we performed KEGG enrichment analysis and selected the top 20 pathways for visualization based on the p value to plot bubble and Sankey diagrams (**Figures 3C, D**). The results revealed that the most prominent pathway among these 20 pathways was the cancer pathway. In addition, PI3K- Akt signaling pathway, MAPK signaling pathway, ErbB signaling pathway and Ras signaling pathway were also involved.

### 3.4 Expression, prognostic levels and diagnostic value of key genes

**Figure 4:**
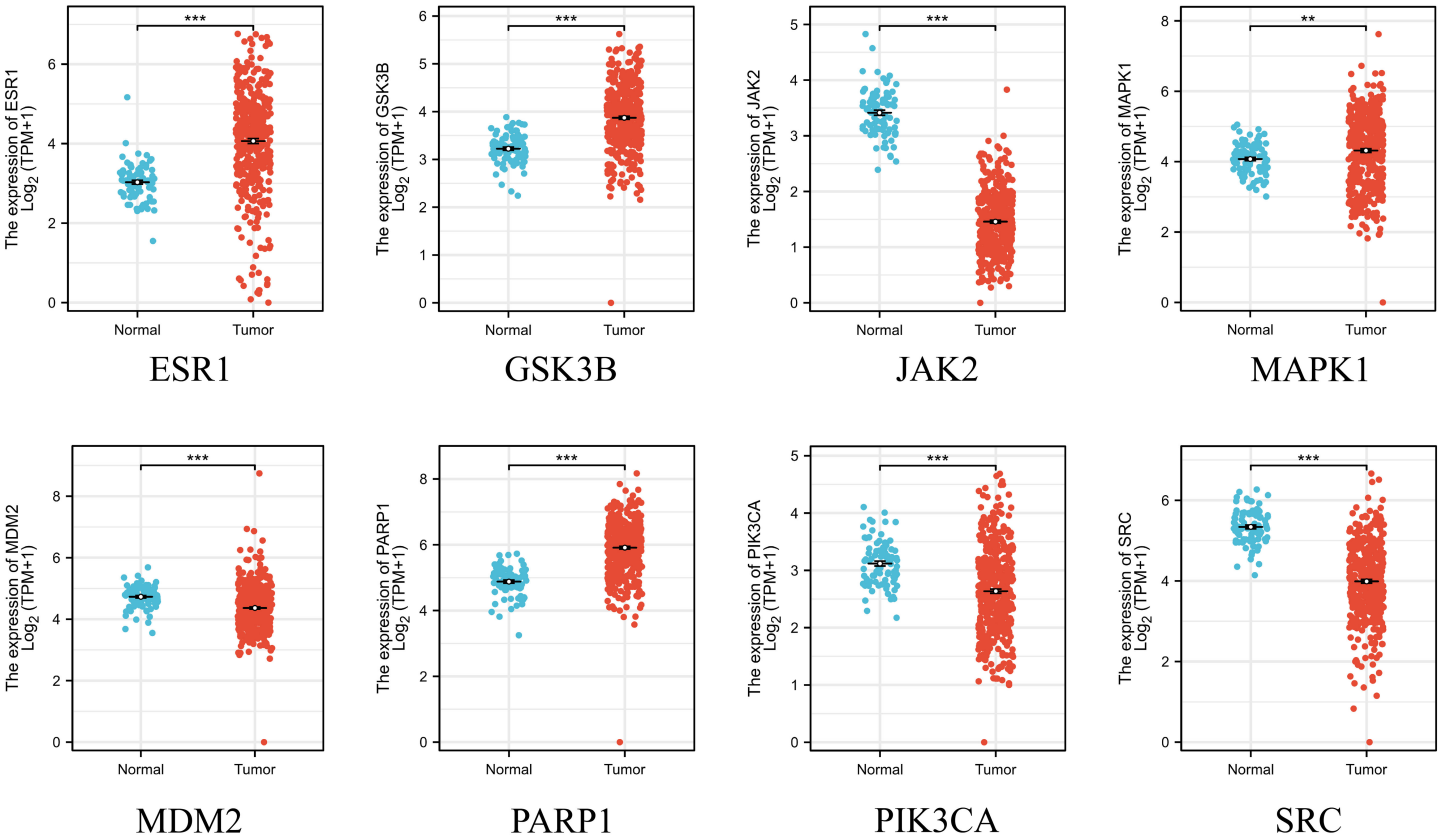
Integration of TCGA database and GTEx database on the expression of 8 key genes in ovarian cancer and normal ovarian tissues. **p < 0.01 and ***p < 0.001 vs. normal tissue.

**Figure 5:**
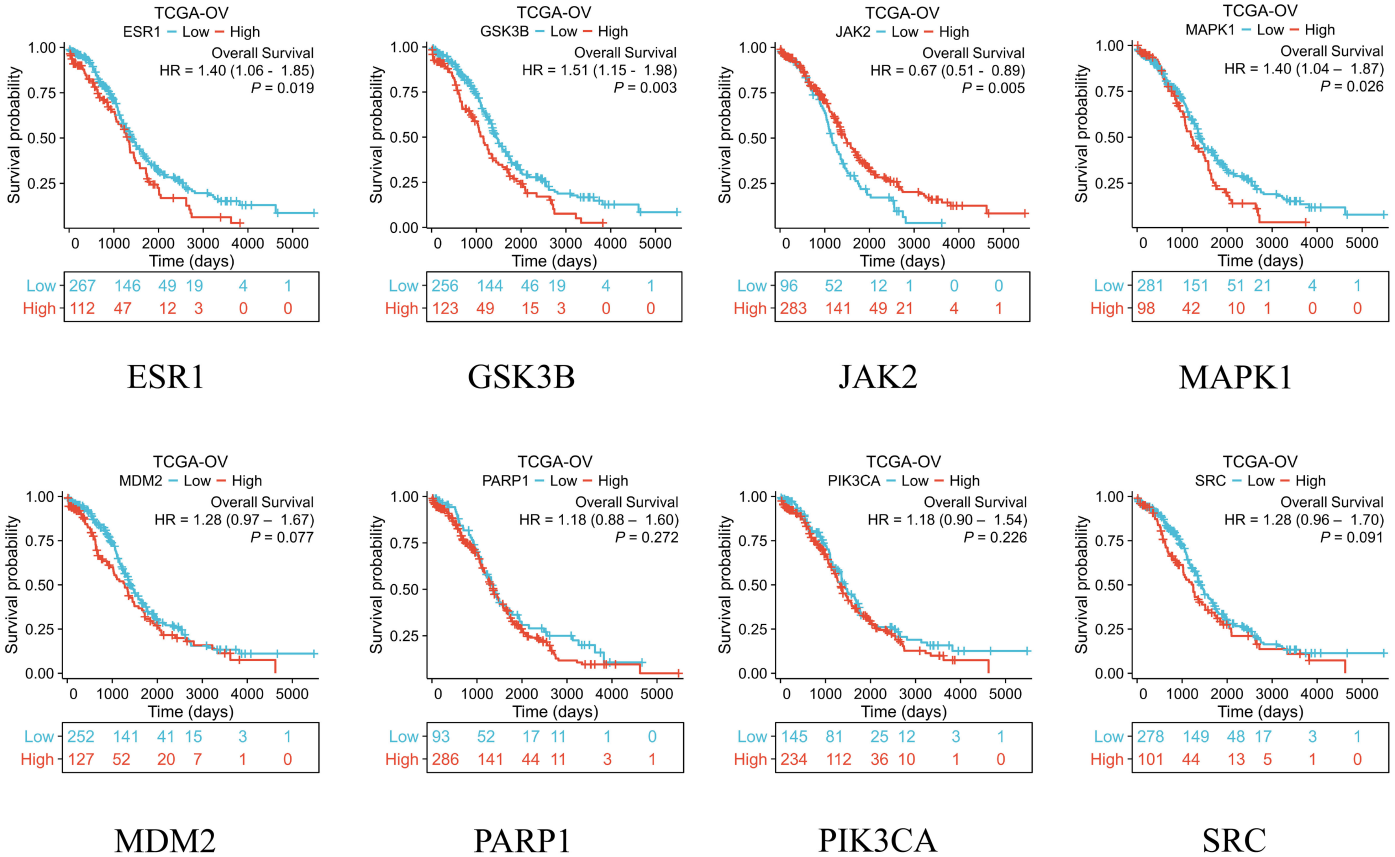
The TCGA database was used to analyze the overall survival of eight key genes.

**Figure 6:**
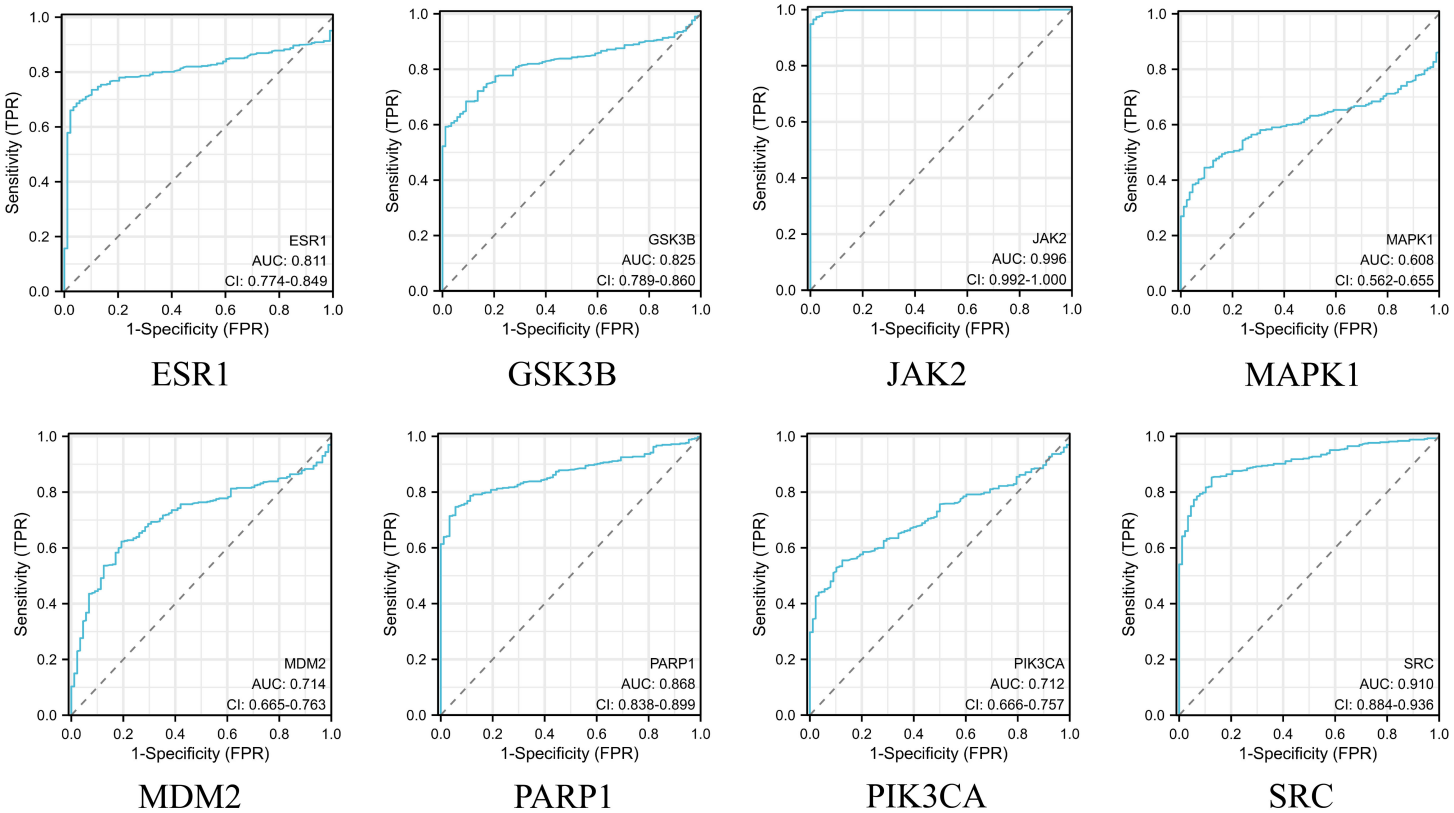
Integration of the TCGA database and the GTEx database to assess the diagnostic value of 8 key genes.

After obtaining eight key genes by network pharmacological analysis, we analyzed their clinical significance by performing differential analysis (**Figure 4**), Kaplan-Meier analysis (**Figure 5**), and ROC diagnostics (**Figure 6**). The expression of key genes between tumor tissues and normal tissues based on both TCGA and GTEx databases were significant. Kaplan-Meier results showed that only ESR1, JAK2, GSK3B, and MAPK1 were statistically significant (P<0.05). ROC diagnostic results showed better diagnostic performance of PARP1, ESR1, JAK2, GSK3B, and SRC (AUC>0.8). Therefore, we obtained the core genes JAK2, GSK3B, ESR1.

### 3.5 Molecular docking

**Figure 7:**
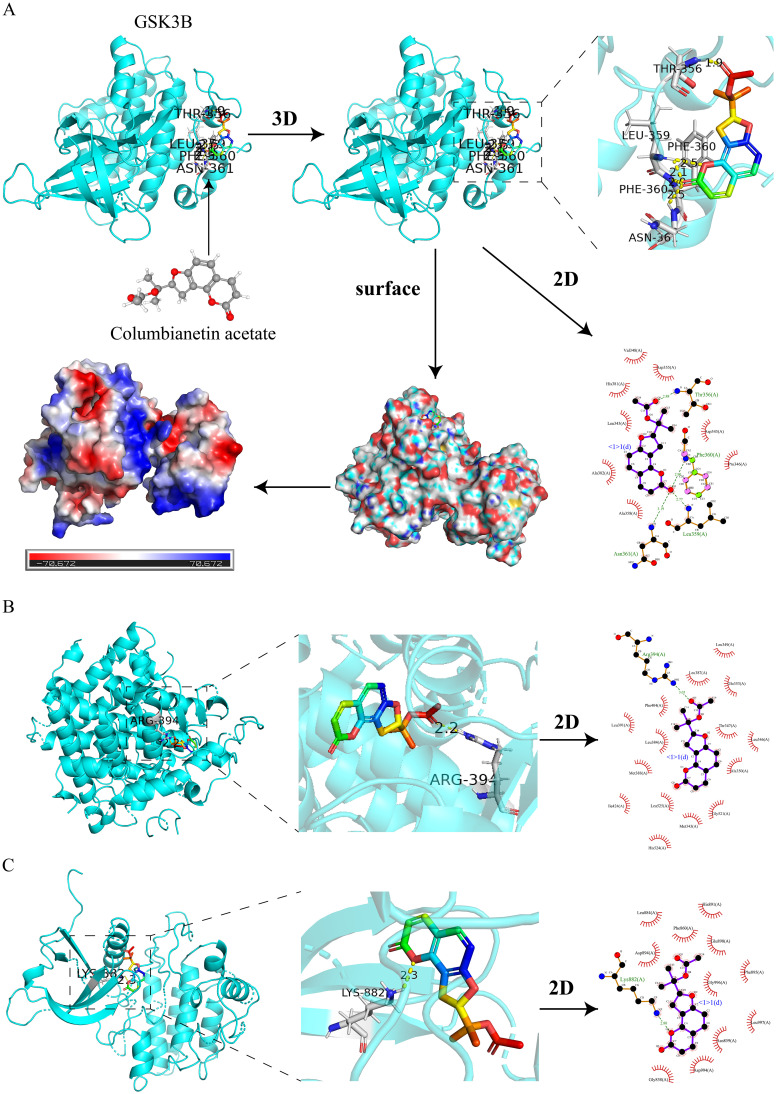
Molecular docking of CE with 3 hub targets. **(A)** Molecular docking of CE with GSK3B is shown in 2D, 3D, surface and energy thermograms. **(B, C)** Molecular docking of CE with JAK2 and ESR1 are shown in 2D and 3D.

To validate the predictions of network pharmacology, we used molecular docking to assess the binding affinity between CE and targets. The lower the binding energy, the more stable the binding between the ligand and the receptor. Normally we consider a binding energy < -5 kcal/mol to indicate good binding between the ligand and the receptor. The docking results showed that the binding energies of CE with GSK3B, JAK2 and ESR1 were -7.41 kcal/mol (**Figure 7A**), -7.3 kcal/mol (**Figure 7B**) and -7.21 kcal/mol (**Figure 7C**), respectively, which indicated that CE had good binding ability with GSK3B, JAK2and ESR1. The detailed information is shown in **Table 1**. It is suggested that these targets may be potential binding targets for CE.

**Table 1:** Binding energy of CE to 3 hub targets.

Target	PDB ID	Bonding energy (kcal/mol)
GSK3B	7B6F	-7.41
ESR1	7NFB	-7.21
JAK2	8BXH	-7.3

### 3.6 KEGG predicts the signaling pathway

**Figure 8:**
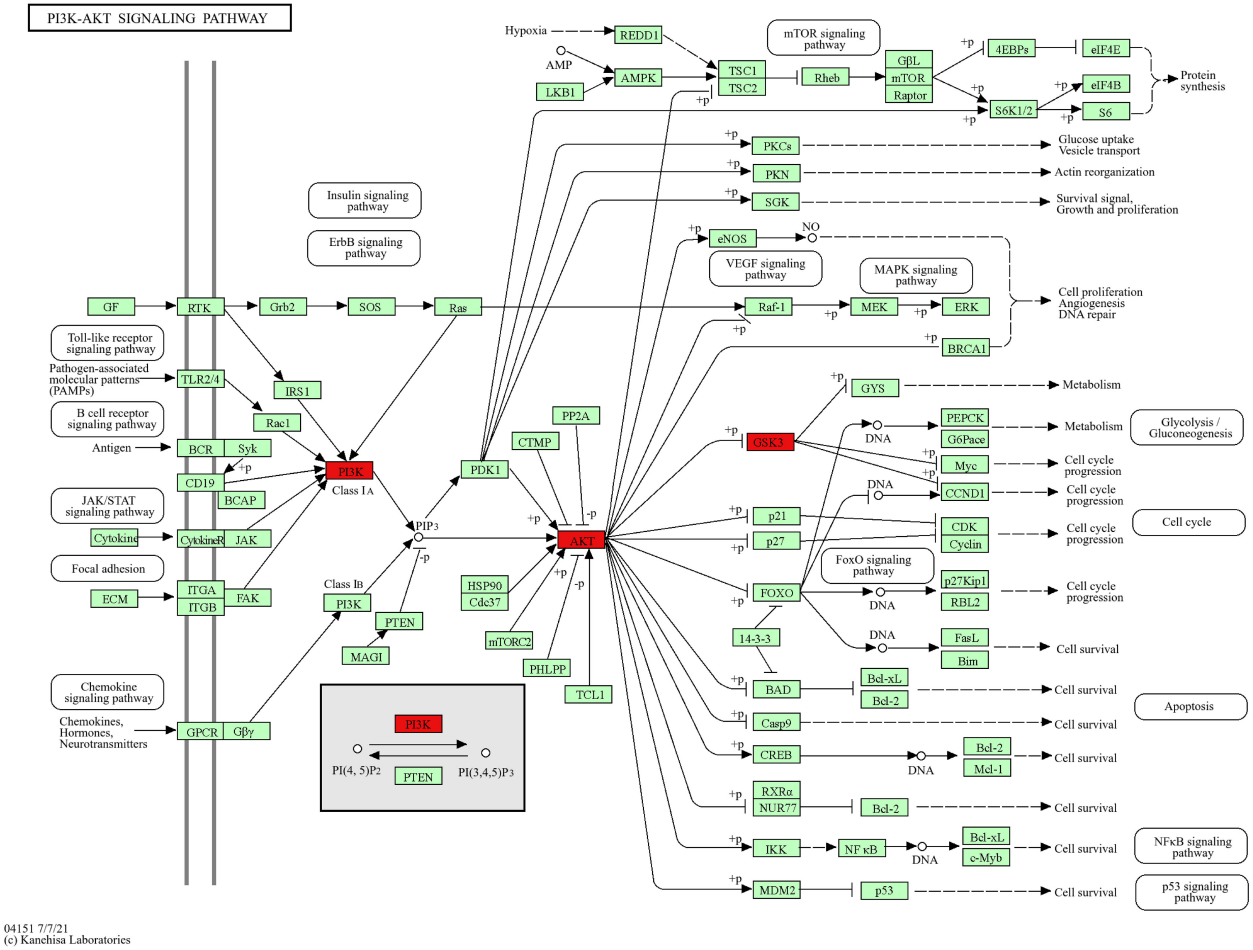
KEGG website to retrieve a map of the key pathways of CE action in ovarian cancer. PI3K/AKT signaling pathway, core targets are marked in red.

The core pathway PI3K/AKT signaling pathway was searched through the KEGG website, and the core target GSK3B is located downstream of the PI3K/AKT signaling pathway (**Figure 8**). Therefore, we predicted that CE might play a role in ovarian cancer by regulating the PI3K/AKT/GSK3B signaling pathway.

### 3.7 CE inhibited ovarian cancer cells proliferation

**Figure 9:**
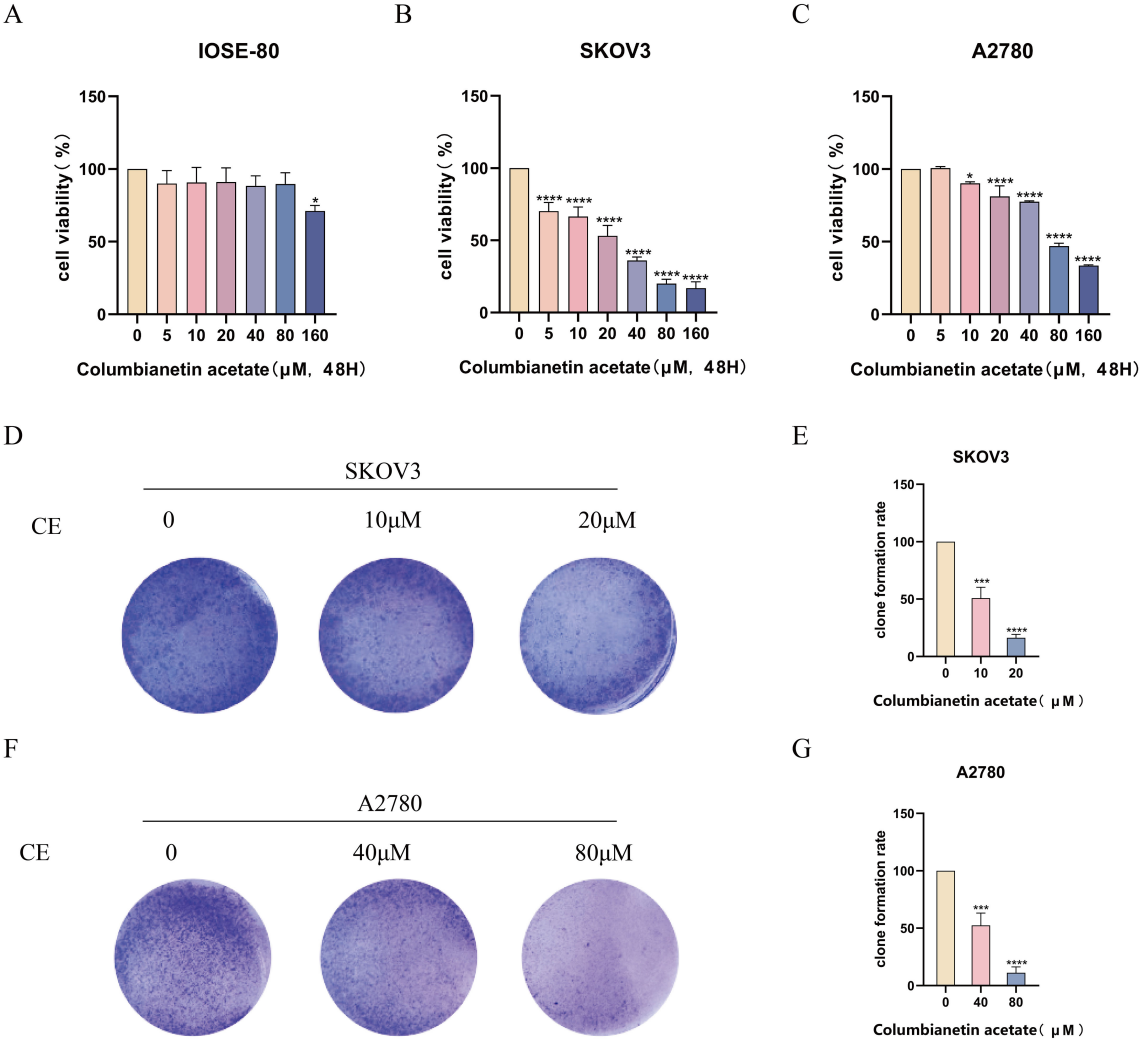
CE inhibited the proliferation of ovarian cancer cells. **(A–C)** The effect of CE on the viability of IOSE-80, SKOV3 and A2780 cells was detected by CCK8 assay. **(D–G)** SKOV3 and A2780 cells were treated with CE for colony formation assay and stained with crystal violet. *p < 0.05, ***p < 0.001 and ****p < 0.0001 and vs. control group.

To evaluate the effect of drugs on ovarian cancer cells proliferation, we performed cell viability assays and colony formation assays using SKOV3 and A2780 cells. CCK-8 results showed that with the increase of CE concentration, there was almost no significant change in cell viability of IOSE-80 cell, and the cell activity of SKOV3 and A2780 cells decreased in a time- and dose-dependent manner, which indicated that CE was almost non-toxic to normal ovarian epithelial cells and inhibitory to ovarian cancer cells (**Figures 9A–C**). Meanwhile, the IC50 values of SKOV3 and A2780 cells at 48 h were 12.22 μM and 41.90 μM. Therefore, in subsequent experiments, we used 10 μM and 20 μM of CE to treat SKOV3 cells and 40 μM and 80 μM of CE to treat A2780 cells, respectively. In addition, colony formation assay results showed that the number of colonies decreased significantly with increasing CE concentration (**Figures 9D–G**). These data suggest that CE can inhibit the proliferation of ovarian cancer cells with little or no toxic effect on normal ovarian epithelial cells.

### 3.8 CE induced apoptosis in ovarian cancer cells

**Figure 10:**
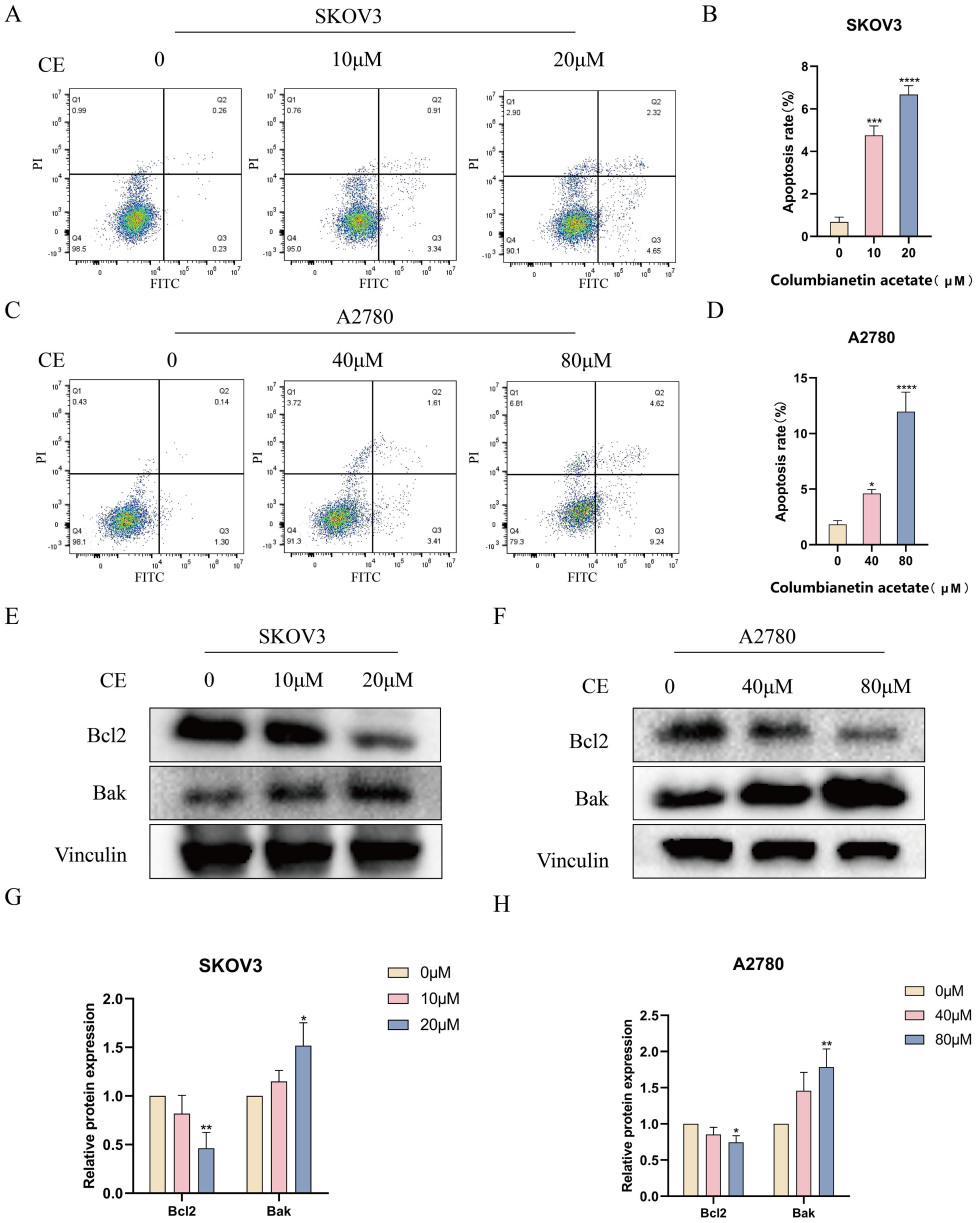
CE promoted apoptosis in ovarian cancer cells. **(A–D)** After 48h of CE action on SKOV3 and A2780 cells, apoptosis was detected by Annexin V/PI double staining. **(E–H)** Expression levels of Bcl2 and Bak proteins in A2780 and SKOV3 cells after CE treatment using western blotting assay. *p < 0.05, **p < 0.01, ***p < 0.001 and ****p < 0.0001 vs. control group.

To investigate whether CE could affect the apoptosis of ovarian cancer cells, Annexin V-FITC/PI double staining was performed after incubating the cells with different concentrations of CE for 48 h. The results showed that the apoptosis rate of cells in the dosing group was significantly higher than that of the control group (**Figures 10A–D**). In addition, 48 h after CE action on ovarian cancer cells, Western blot analysis showed that the expression level of Bak was significantly increased, while the expression level of Bcl-2 was significantly decreased (**Figures 10E–H**). These findings indicated that CE can induce apoptosis in ovarian cancer cells.

### 3.9 CE inhibited ovarian cancer cells invasion and migration

**Figure 11:**
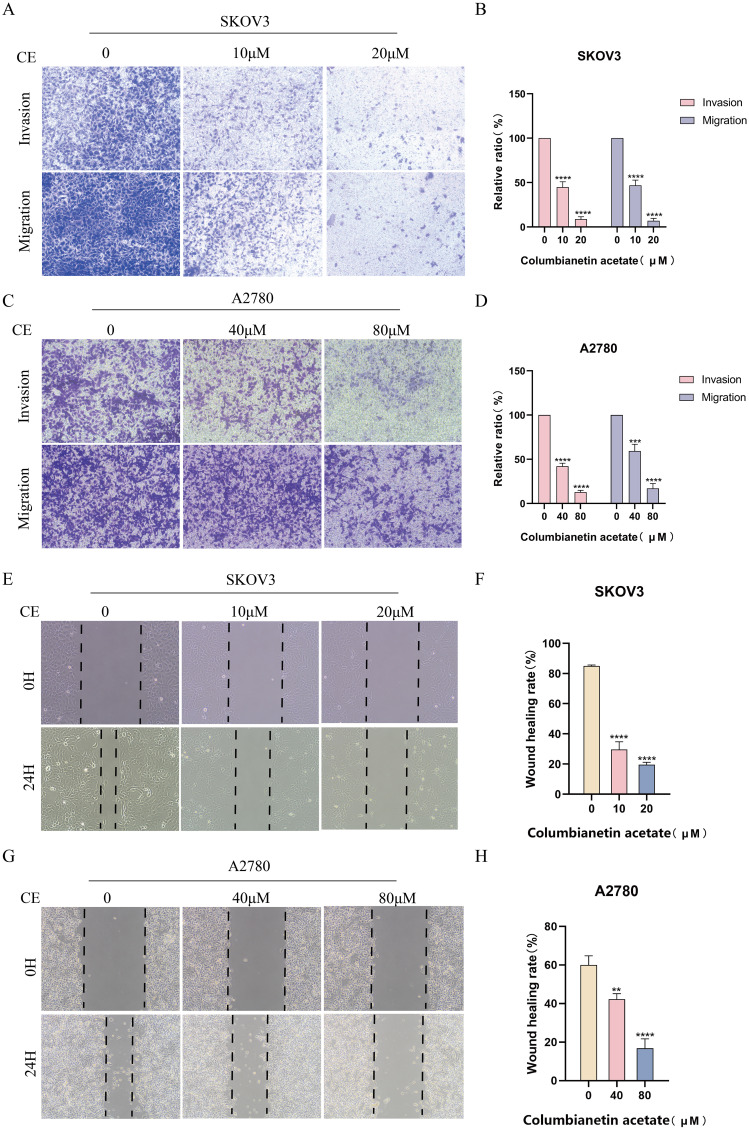
CE inhibited invasion and migration of ovarian cancer cells. **(A–D)** Effect of CE on the migration and invasion ability of SKOV3 and A2780 cells using transwell assay. **(E–H)** Effect of CE on the migratory ability of SKOV3and A2780 cells detected using wound healing assay. **p < 0.01, ***p < 0.001 and ****p < 0.0001 vs. control group.

Next, we performed transwell assays and wound healing assays to test that CE plays a role in the invasion and migration of ovarian cancer cells. The results of the transwell assay showed that the rate of invasion and migration was significantly slower in the treatment group with the addition of CE compared to the control group (**Figures 11A–D**). Wound healing experiments demonstrated the same results, with increasing CE concentration, the scratch area at 24 h was higher than that of the control group (**Figures 11E–H**). Collectively, these findings indicate that CE could significantly inhibit the invasion and migration of ovarian cancer cells.

### 3.10 CE inhibited EMT in ovarian cancer cells

**Figure 12:**
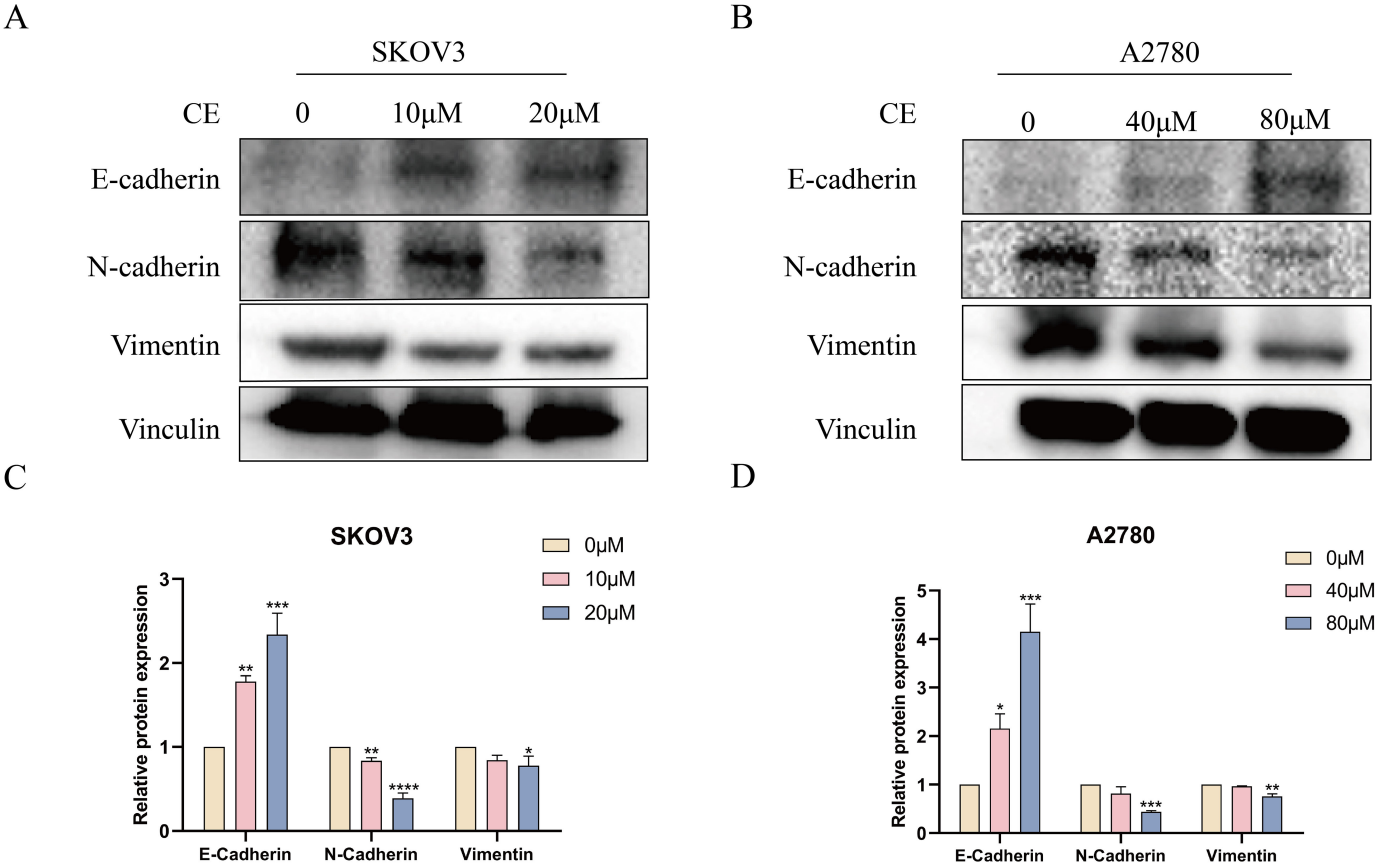
CE inhibited the EMT process in ovarian cancer cells **(A–D)** The expression levels of E-cadherin, N-cadherin and Vimentin proteins in A2780 and SKOV3 cells after CE treatment were examined using western blotting assay. *p < 0.05, **p < 0.01, ***p < 0.001 and ****p < 0.0001 vs. control group.

In addition to detecting invasion and migration, we also detected the expression of EMT markers, namely E-cadherin, N-cadherin and Vimentin. Western blot results showed a significant increase in E-cadherin expression and a significant decrease in N-cadherin and Vimentin protein expression with increasing CE concentration compared to the control group (**Figures 12A-D**). Together, these findings clearly indicated that CE can inhibit the EMT process in ovarian cancer.

### 3.11 CE may act on ovarian cancer cells by regulating the PI3K/AKT/GSK3B pathway

**Figure 13:**
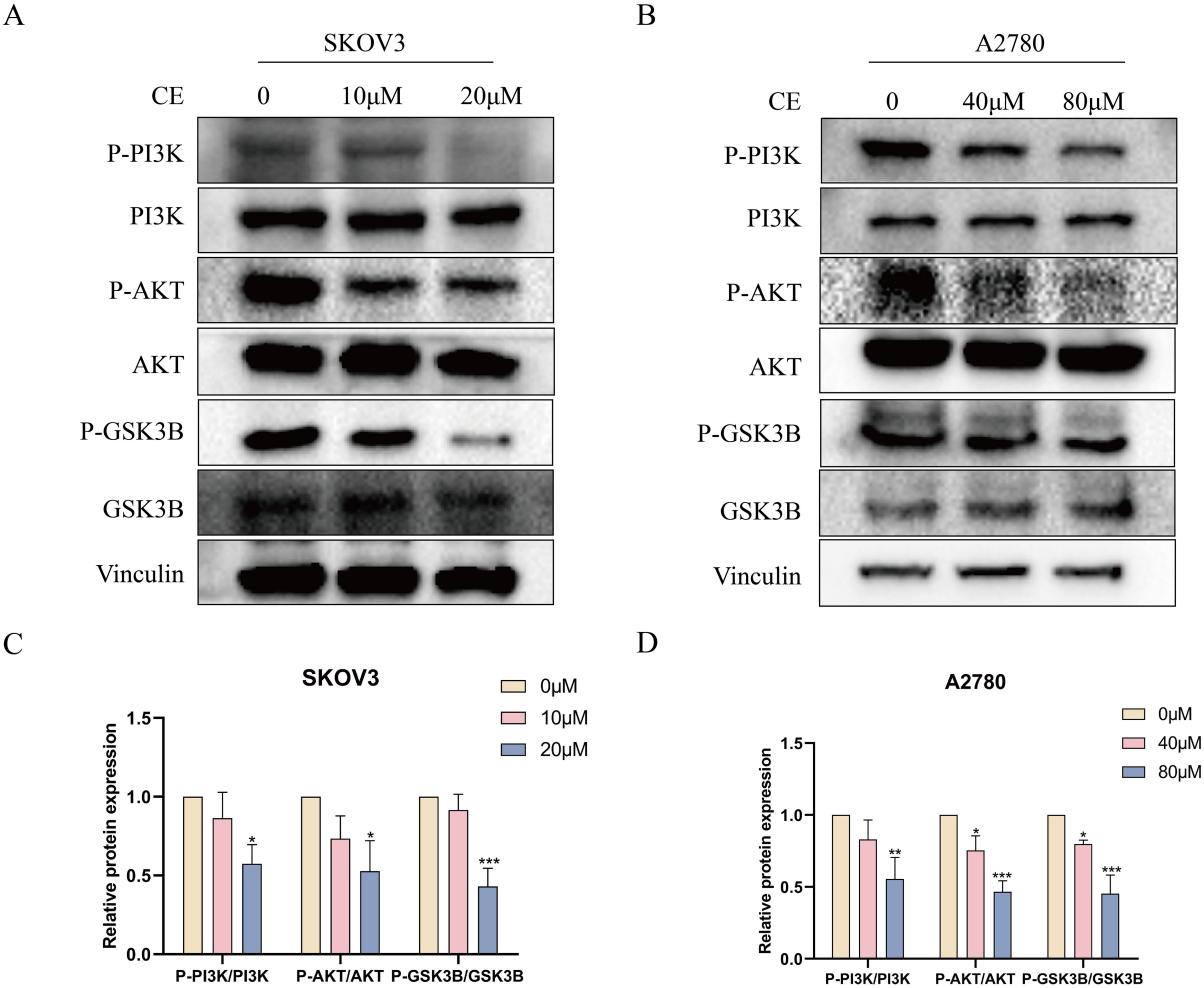
CE inhibited the PI3K/AKT/GSK3B signaling pathway in ovarian cancer cells. **(A–D)** The expression levels of P-PI3K/PI3K, P-AKT/AKT and P-GSK3B/GSK3B proteins in A2780 and SKOV3 cells after CE treatment were examined using western blotting assay.*p < 0.05, **p < 0.01 and ***p < 0.001 vs. control group.

KEGG pathway analysis predicted that CE might affect ovarian cancer by regulating the PI3K/AKT/GSK3B pathway. To verify this, we examined the expression of key proteins of the pathway after 48 h of CE treatment. Western blot results showed that the expression of P-PI3K/PI3K, P-AKT/AKT and P-GSK3B/GSK3B were reduced in ovarian cancer cells in the CE-treated group compared with the control group (**Figures 13A-D**). Therefore, we suggest that CE may play an inhibitory role in ovarian cancer by inhibiting the PI3K/AKT/GSK3B signaling pathway.

## 4 Discussion

Ovarian cancer is a malignant tumor with poor prognosis and high recurrence rate (16, 17), which seriously threatens women’s health and life. One of the biggest obstacles to treating ovarian cancer is the problem of chemotherapy resistance (18). Chinese medicine chemicals are more effective in treating diseases due to their multi-component, multi-targeted nature and lower side effects when compared to chemical medications and biopharmaceuticals (19). Angelica sinensis is a traditional Chinese herb with anti-inflammatory, anti-tumor and sedative properties (20). Columbianetin acetate, as one of the main active constituents of Angelica sinensis, has anti-inflammatory properties and a high small intestinal absorption rate (21). However, it remains poorly studied in tumors, and this study focuses on exploring the mechanism of action of CE in ovarian cancer, providing new insights into the role of CE in the treatment of tumors. In the current study, we used network pharmacology, bioinformatics and molecular docking techniques to screen the core genes and pathways of CE action in ovarian cancer, and found that CE induced apoptosis and inhibited metastasis of ovarian cancer cells by *in vitro* experiments.

Apoptosis is a form of programmed cell death, a process of gene-regulated, self-induced cellular death. It can be classified into intrinsic and extrinsic apoptotic pathways, with its mechanisms involving numerous signaling pathways (22). Since apoptosis plays a key role in the pathogenesis of many diseases, understanding the mechanisms of apoptosis is crucial for cancer treatment (23). The endogenous apoptotic pathway is an important mechanism of apoptosis and is usually triggered by intracellular stress (e.g., oxidative stress, DNA damage, cellular nutrient deficiencies, etc.) (24). The Bcl-2 family includes pro-apoptotic proteins (e.g., Bax, Bak) and inhibitory proteins (e.g., Bcl-2, Bcl-xL), and pro-apoptotic proteins are activated under stressful conditions, leading to disruption of the permeability of the outer mitochondrial membrane (25). This prompts the release of cytochrome c into the cytoplasm, where cytochrome c binds Apaf-1, forming apoptotic vesicles that activate caspase-9, which further activates executive caspases such as caspase-3, ultimately leading to cell death (26, 27). After CE acted on ovarian cancer cells, apoptotic cells were detected using flow cytometry, which showed that the number of apoptotic cells increased with the increase of drug concentration compared with the control group. At the same time, we detected the expression of apoptotic proteins by western blot assay, and found that the expression level of Bcl-2 was significantly reduced after drug action, while Bak showed the opposite trend. These results revealed that CE could induce apoptosis in ovarian cancer cells.

Prevention of tumor metastasis can go some way to controlling deaths caused by tumors (28). Epithelial-mesenchymal transition (EMT) plays an important role in tumor metastasis and is an important biological process by which malignant tumor cells derived from epithelial cells acquire the ability to migrate and invade (29–31). EMT is characterized by a reduction in adhesion junctions and apical-basal polarity, which ultimately promotes cell motility and invasion (32). EMT promotes tumor invasion and metastasis mainly by altering the microenvironment, leading to tumor growth, invasion, metastasis and angiogenesis (33). Research indicates the existence of numerous signaling pathways methods engaged in EMT, encompassing transformative growth factor beta (TGF-β), reliant on SMAD/independent, Wnt/β-catenin, Matrix, PI3K/AKT/mTOR, and AKT/GSK3β pathways of catenin signaling (34–36). Our present study demonstrated that CE could inhibit the viability, invasion and migration of ovarian cancer cells by CCK8, scratch and transwell assays. Moreover, it was found by western blot assay that the action of CE significantly elevated the expression of E-Cadherin protein, while the protein expression of N-Cadherin and Vimentin was reduced, which indicated that CE could inhibit the EMT process of ovarian cancer cells. In summary, our findings show that CE can inhibit the growth and metastasis of ovarian cancer cells.

Based on the KEGG enrichment results, after CE action, PI3K/Akt, MAPK and Ras signaling pathways intertwine with each other to form a complex signaling network, which affects the biological behaviors of ovarian cancers. CE may promote the anticancer effects by regulating the interactions of these pathways. Subsequently, we validated the PI3K/AKT signaling pathway, a key pathway for CE action in ovarian cancer, and found the core target GSK3B as downstream of PI3K/AKT at the KEGG website. Furthermore, PI3K/AKT/GSK3B is aberrant in a wide range of cancers and has been shown to play a key role in cancer cell proliferation, migration, invasion and apoptosis (37). Based on this we performed western blot experiments, which showed that the expression of P-PI3K/PI3K, P-AKT/AKT and P-GSK3B/GSK3B decreased with increasing concentrations of CE. These results suggest that CE can inhibit ovarian cancer growth by inhibiting the PI3K/AKT/GSK3B signaling pathway. This study presents a new perspective on the molecular mechanism of traditional Chinese medicine in the treatment of ovarian cancer, and we need to perform *in vivo* experiments to further validate it. However, this study still has some shortcomings. First, collecting predictive targets from publicly available databases requires more convincing prospective studies to confirm our findings. In addition, the validation of the pathway could be further verified by adding inhibitors or activators of the PI3K pathway. Finally, *in vivo* experimental validation remains to be performed. Nevertheless, our findings suggest that CE may be a promising candidate for the treatment of ovarian cancer, providing a theoretical basis for clinical therapy.

## 5 Conclusion

In conclusion, the present study demonstrated that Columbianetin acetate could inhibit the proliferation and metastasis of ovarian cancer and induce apoptosis by inhibiting the PI3K/AKT/GSK3B pathway. It provided a new direction for the treatment of ovarian cancer and a new idea for future clinical translational research.

## Data Availability

The original contributions presented in the study are included in the article/supplementary material. Further inquiries can be directed to the corresponding authors.
